# Swirling Instability of the Microtubule Cytoskeleton

**DOI:** 10.1103/PhysRevLett.126.028103

**Published:** 2021-01-15

**Authors:** David B. Stein, Gabriele De Canio, Eric Lauga, Michael J. Shelley, Raymond E. Goldstein

**Affiliations:** 1 Center for Computational Biology, Flatiron Institute, 162 5th Avenue, New York, New York 10010, USA; 2 Department of Applied Mathematics and Theoretical Physics, Centre for Mathematical Sciences, University of Cambridge, Cambridge CB3 0WA, United Kingdom; 3 Courant Institute, New York University, 251 Mercer Street, New York, New York 10012, USA

## Abstract

In the cellular phenomena of cytoplasmic streaming, molecular motors carrying cargo along a network of microtubules entrain the surrounding fluid. The piconewton forces produced by individual motors are sufficient to deform long microtubules, as are the collective fluid flows generated by many moving motors. Studies of streaming during oocyte development in the fruit fly *Drosophila melanogaster* have shown a transition from a spatially disordered cytoskeleton, supporting flows with only short-ranged correlations, to an ordered state with a cell-spanning vortical flow. To test the hypothesis that this transition is driven by fluid-structure interactions, we study a discrete-filament model and a coarse-grained continuum theory for motors moving on a deformable cytoskeleton, both of which are shown to exhibit a *swirling instability* to spontaneous large-scale rotational motion, as observed.

A striking example of fluid-structure interactions within cells [1] occurs in oocytes of the fruit fly *Drosophila melanogaster* [2]. These develop over a week from a single cell through repeated rounds of cell division, differentiation, and growth, ultimately reaching hundreds of microns across. This pathway has been divided into 14 stages, and it is in stages 9–11, at days 6.5–7 [3], that fluid motion is most noticeable. In stage 9 (Fig. 1), microtubules (MTs) reach inward from the periphery, forming a dense assembly along which molecular motors (kinesins) move at tens of nm/sec, carrying messenger ribonucleic acids and other nanometric particles. This motion entrains the surrounding fluid, producing cytoplasmic streaming [4,5] that can be visualized several ways: in bright field by the motion of endogenous particles [6–8], via their autofluorescence [9,10], and through a combination of particle image velocimetry and fluorescently labeled microtubules [11–13]. Previous work [7,11] revealed that these initial flows exhibit transient, recurring vortices and jets whose correlation length is a fraction of the cell scale, with no long-range order. But by stage 11, a dramatic reconfiguration of the cytoskeleton occurs, coincident with the appearance of a cell-spanning vortex [6,7,10,14].

Kinesins move from *minus* ends of microtubules (attached to the oocyte periphery) to *plus* ends (free in the interior). Transport of cargo through the network depends on motor-microtubule binding [16,17] and the mesh architecture [18,19]. As a motor pulls cargo toward the plus end, the filament experiences a localized minus-end-directed compressive force, as in Euler buckling. For a filament of length *L* and bending modulus *A* [20], the buckling force is *π*^2^*A*/4*L*^2^ ~ 50 pN*/L*^2^, where *L* is measured in microns. Thus, a kinesin’s force of several pN [21] can buckle MTs 10–40 *μ*m long.

The coupled filament-motor problem is richer than Euler buckling because a motor exerts a “follower force” [22] that is aligned with the filament. This feature breaks the variational structure of the problem and induces a filament pinned at its minus end to oscillate at zero Reynolds number [23–25]. By exerting a force on the fluid, a motor induces long-range flows which can further deform filaments [26,27].

It has been hypothesized [10,14] that the transition from disordered flows to a single vortex in stage 11 is a consequence of fluid-structure interactions, facilitated by a decrease in cytoplasmic viscosity that accompanies the disappearance of a coexisting network of the biopolymer f-actin. Here, through a combination of direct computations on the coupled filament-flow problem [24] and studies of a continuum theory for dense filament suspensions [28], we confirm this hypothesis by showing the existence of a *swirling instability* of the cytoskeleton.

Swirling can be understood in a simplified model of the oocyte: a rigid sphere of radius *R* containing a fluid of viscosity *μ*, with *N* elastic filaments reaching inward from clamped attachment points equally spaced around the equator. A slice in the filament plane [Fig. 2(a)] appears like the confocal slice in Fig. 1. The filaments have a radius *r*, a constant length *L*, bending modulus *A*, and a uniform line density *f* of follower forces [Fig. 2(b)].

Some comments are in order. Although free MTs have a complex dynamics of growth and decay, recent evidence [29] for “superstable” cortically bound MTs in stages displaying unidirectional streaming justifies the constant-length approximation. As the exact nature of cortical MT binding is unclear, we make the simplest assumption of orthogonal clamping to a rigid cortex. Finally, the model is agnostic regarding the transported cargo, provided the resultant forces on the fluid and fiber are equal and opposite, and aligned with the fiber [29].

Microtubules are the quintessential slender bodies [30] of biophysics, with aspect ratios *ε* = *r*/*L* of 𝒪 (10^−3^). As their self-interactions are weak, we use local slender-body theory [31,32] to obtain the dynamics. In an arclength parametrization *s*, the *j*th filament **r**^*j*^(*s, t*) evolves as

(1) $$\eta \left(r_{t}^{j}-U^{j}\right)=\left(I+r_{s}^{j}r_{s}^{j}\right)\left[-Ar_{4s}^{j}+{\left({\text{Λ}}^{j}r_{s}^{j}\right)}_{s}-fr_{s}^{j}\right],$$

where $r_{s}^{j}$ is the unit tangent, *η* = 8*πμ*/*c*, with *c* = ❘ln(*eε*^2^)❘, and the Lagrange multiplier Λ^*j*^ enforcing inextensibility obeys a second-order partial differential equation [33]. In the background flow **U**^*j*^ / **u**^*j*^ *+* **u**^*i*→*j*^ *+* **v**^*i*→*j*^, **u**^*j*^ is that produced by the motors on *j*, **u**^*i*→*j*^ is due to the motors on *i* ≠ *j*, and **v**^*i*→*j*^ is due to motion of filaments *i* ≠ *j*. For example, the flow induced at **x** by motors dragging cargo along the *j*th fiber is ${\int \;}_{0}^{L}dsfr_{s}^{j}(s)\cdot G\left[x-r^{j}(s)\right]$ (see the Supplemental Material [34,35]), with **G** the Green’s function for the interior of a no-slip sphere [36]. Filament clamping at the sphere implies that **r**^*j*^(0; t) remains fixed and $r_{s}^{j}(0,t)$ is the local inward sphere normal. The free end is torque and force free: $r_{ss}^{j}(L,t)=r_{sss}^{j}(L,t)=\text{Λ}(L,t)=0$.

A single fiber clamped at a flat wall displays a super-critical Hopf bifurcation which, expressed in terms of the dimensionless motor force *σ* ≡ *fL*^3^/*A*, occurs at σ* ≃ 124.2, beyond which the filament exhibits steady oscillations with amplitude $\sim \sqrt{\sigma -\sigma^{*}}$ [24]. When several filaments interact within the sphere (2c), they also oscillate, but with their motions synchronized in phase like eukaryotic flagella [37]. The dynamical model (1) contains two ingredients often found necessary for such synchronization [38,39]: hydrodynamic interactions and the ability of a filament to change shape and thereby adjust its phase in response to those flows.

As the filament density and motor strength are increased, we find the swirling instability: transition to a *steady* state of bent filaments whose free ends are nearly parallel to the wall [Fig. 2(d)]. This bending is maintained by motor-induced azimuthal flows that generate drag along the distal ends of filaments and thus a torque countering bending torques nearer the base. As with any such spontaneous symmetry breaking, initial conditions dictate the choice between equivalent left- and right-handed configurations. This transition is reminiscent of self-organized rotation of cytoplasmic droplets extracted from plants [40] and the spiral vortex state of confined bacterial suspensions [41], both modeled as force dipole suspensions [42–44]. A “locked-curvature” regime of free, axially driven filaments, reminiscent of the bent MTs in the swirling state, has also been observed [45].

While direct computations on denser arrays of discrete filaments are possible [46], cortically bound oocyte MTs are so tightly packed, with an interfiber spacing *δ* ≪ *L* [10–13], that a continuum approach is justified. The description we use [28], in which microtubules form an anisotropic porous medium, is based on the map **X** = **r**(***α***), where the Lagrangian coordinate ***α*** = (*α,s*) encodes the location *α* of the minus ends of the microtubules and arclength *s*. In a system of units made dimensionless by *L* and elastic relaxation time *ηL*^4^/*A*, we obtain a continuum version of (1),

(2) $$r_{t}-{u|}_{r(\alpha )}=\left(I+r_{s}r_{s}\right)\cdot \left[-r_{ssss}+{\left(\text{Λ}r_{s}\right)}_{s}-\sigma r_{s}\right].$$

The fluid velocity **u** arises from the force distribution along the filaments and is evaluated at the Eulerian position **x** according to an inhomogeneous Stokes equation,

(3) $$-\nabla^{2}u+\nabla p={\chi_{\text{mt}}\rho \left[J^{-1}\left[-r_{ssss}+{\left(\text{Λ}r_{s}\right)}_{s}\right]\right]|}_{r^{-1}(x)},$$

subject to the incompressibility constraint ∇ · **u** = 0. The indicator function *χ*_mt_ is supported where the MT array is present [Fig. 3(a)]. Here, *ρ* = 8*πρ*_0_*L*^2^/*c* is the rescaled areal number density of microtubules, expressible as *ρ* = *ϕ* (*L*/*δ*)^2^, where the constant *ϕ* depends only on the MT slenderness and packing geometry at the wall; *ϕ* ≈ 4 when *c* ≈ 10 and the MTs are hexagonally packed. The quantity 𝒥 det [∂**r**/∂**α**] measures the change in microtubule density due to deformations of the array; 𝒥^−1^ increases as fibers move closer together.

The simplest geometry is an infinite planar array of MTs with the same boundary conditions as in the discrete model [Fig. 3(a)], and with no-penetration and zero-tangential stress conditions on the fluid a distance *H* above the wall. For dynamics homogeneous along *x*, the fluid flow is unidirectional and constant above the MTs, so *H* plays no role. Nonlinear computations [47] reveal both oscillatory dynamics and the emergence of steady streaming. Figure 3(b) shows the dynamics when *ρ* = 4.65 and *σ* = 70: self-sustaining oscillations of the MT array are observed, similar to those in Fig. 2(c). Note that while Fig. 3(b) shows only a single filament, it represents the common dynamics of *all* of the collectively beating filaments in the array. When *σ* is decreased to ≈ 39, the MT array deforms and stabilizes into a steady bent state [Fig. 3(c)]. This is the continuum version of the swirling transition, with dynamics similar to the discrete case.

An equilibrium of the system occurs when filaments are aligned straight along *z*, with **u** = 0 and Λ = −*σ* (1 − *z*). For *σ* > 0, the motor force is compressive and buckling may occur. A small transverse perturbation in fiber shape of the form $r_{s}=\hat{z}+\epsilon g(z)\hat{x}(\epsilon \ll 1)$ evolves as

(4) $$g_{t}=-g_{zzzz}-\sigma {\left[(1-z)g_{z}\right]}_{z}+\rho \left[\sigma (1-z)g+g_{zz}\right].\;$$

The first two terms are like those of an elastic filament under an aligned gravitational field, with a linearly varying tension [48,49]. The third is fiber forces filtered through the nonlocal Stokes operator, capturing hydrodynamic interactions within the fiber array (hence the *ρ* prefactor). Here, the simplicity of the flow is such that inverting the Stokes equations does not lead to the typical global coupling. The term *ρg*_*zz*_ captures the additional resistance to bending from flow: if a MT bends, it moves the nearby fluid, bending other MTs; the term *ρσ*(1 – *z*)*g* is destabilizing: if a MT remains straight, it must resist fluid motions generated by surrounding MTs.

The coarse-grained model in planar geometry reproduces the behavior of the discrete-filament model. To capture features of the oocyte geometry—its convex shape and confined hydrodynamic interactions—we extend the analysis to a cylindrical domain, where the no-flow steady state is an array of MTs pointing straight inward. Figure 4 shows the results of a linear stability analysis for an experimentally relevant ratio of cylinder diameter to MT length of 10:1. For *ρ* ≪ 1, the continuum model behaves like isolated fibers with negligible collective fluid entrainment. For small *σ*, straight fiber arrays are stable (regions I and II, with region II having oscillatory decay to equilibrium), but with increasing *σ* there is a Hopf bifurcation to a state that nonlinear simulations show has oscillations [cf. Fig. 3(b)]. For *ρ* ≳ 2.8 (*δ* ≲ 1.2*L*), a new region of instability (IV) appears, with real, positive eigenvalues; nonlinear simulations show this leads to collective MT bending and swirling flows [cf. Fig. 3(c)]. The structure of these transitions is independent of the degree of confinement [34].

Figure 4(b) shows a nonlinear computation of the transition to swirling in region IV. The upper inset shows the development of the instability, with successive MTs bending over to form a dense canopy above their highly curved bases. Once steady, the concentrated motor forces within the canopy are azimuthally aligned, almost a *δ*-function a distance ~*L*/4 above the wall, and drive the large-scale streaming flow. The ooplasmic flow beneath the MT canopy is nearly a linear shear flow, transitioning above to solid body rotation, the solution to Stokes flow forced at a cylindrical boundary.

We now estimate ranges of density and force that are consistent with observed streaming speeds *u* ≈ 100–400 nm/s (Fig. 1 and [14,29]). Taking *L* = 20 *μ*m, *μ* = 1 Pas [11], and *A* = 20 pN *μ*m^2^, we obtain a velocity scale *A*/*ηL*^3^ ≈ 1 nm/s and a force-density scale *A*/*L*^3^ ≈ 2.5 fN/*μ*m. Figure 4(c) shows the speeds computed in region IV. Those with maximum speeds falling in the experimental range lie in the hatched area. Increasing *ρ* only marginally increases streaming speeds, and so to increase flow speed while remaining in region IV requires increasing both *ρ* and *σ*. The minimum value of *ρ* ≈ 20 that is consistent with observed streaming velocities corresponds to *δ* ≲ 0.4*L*, a more stringent constraint than that required for the streaming transition. The force densities consistent with streaming speeds are *f* ~ 0.1–0.6 pN/*μ*m. Speeds on the higher end of the range approach the ≈ 700 nm/s of kinesin-1 under negligible load [21], while cargo speeds on oocyte MTs are 200–500 nm/s [14,29,50]. Assuming a linear force-velocity relation and a stall force of 6 pN [21] give a single motor force of ≈ 2 pN; approximately 1–6 kinesins are needed per 20 *μ*m MT to generate these force densities.

A heuristic argument for the weak dependence of flow speeds on *ρ* views the cytoskeleton as a porous medium of permeability *k* ~ *δ*^2^, in which speed *u* ~ (*k*/*μ)* ∇*p*, where the pressure gradient (force/volume) from motors is *f*/*δ*^2^, yielding *u* ~ *f*/*μ* ~ (*A*/*ηL*^3^)(8*π*/*c*)*σ*, independent of *ρ*. This relationship is surprisingly accurate [34].

When the density *ρ* is sufficiently high, the swirling instability first appears for force densities *σ* substantially smaller than those that induce oscillatory instabilities in a single filament; this transition is driven by additional hydrodynamic destabilization imparted by neighboring fibers [in planar geometry, the term *ρσ*(1 – *z*)*g* in Eq. (4)]. This observation motivates a heuristic argument for the instability, in which a filament is bent by the flow produced by its upstream neighbor, whose distal half is nearly parallel to the wall. Seen from a distance, that bent portion acts on the fluid like a point force [51] **F** ~ (*fL*/2) **r**_*s*_ *L* oriented along its tangent vector (Fig. 5), displaced a distance *h*~ *L*/2 from the surface. Near a no-slip wall, the far-field flow along *x* due to a force $F∥\hat{x}$ a distance *δ* upstream is simple shear [52,53],

(5) $$U(x,z)=\dot{\gamma }z{\hat{e}}_{x},$$

where $\dot{\gamma }=3hF/2\pi \mu \delta^{3}$. Self-consistency requires the magnitude of the force driving the shear be given by the projection of **F** along *x*, so $\dot{\gamma }\to \dot{\gamma }\sin[\theta (L)]$.

The simplest model to illustrate the self-consistency condition is a rigid MT with a torsional spring at its base that provides a restoring torque −*kθ* [Fig. 5(i)]. With *z* (*s*) = *s* cos *θ* and $\eta \hat{n}\;\hat{n}$ ·**U** the local normal force on a segment, the local torque about the point *s* = 0 is $\eta \dot{\gamma }s^{2}{\text{cos}}^{2}\theta$ cos^2^ *θ* which, when integrated along the filament and balanced against the spring torque, yields the self-consistency condition

(6) $$\theta =B\sin\theta \;\cos^{2}\;\theta ,$$

where $B=\eta \dot{\gamma }L^{3}/k$. For *B* < 1 (slow flow or a stiff spring), *θ* = 0 is the only fixed point, while for *B* ≳ 1 two mirror-image swirling solutions appear through a pitchfork bifurcation, *θ*_±_ ≃ [6 (*B* – 1)/7]^1/2^. A structurally similar model has been used to explain cytoplasmic streaming in the *Caenorhabditis elegans* zygote [54].

To study the interplay between filament oscillations and swirling we use (5) in the dynamics (1), where the control parameter for the shear flow is [26,27]

(7) $$M=\frac{\eta \dot{\gamma }L^{4}}{A}\sim \frac{3\sigma }{c}{\left(\frac{\rho }{\phi }\right)}^{3/2},$$

and the second relation uses the estimates above for *F* and *h*. Because a clamped elastic filament behaves like a torsional spring with spring constant *k* = *A*/*L*, we see consistency with the parameter *B* above. A numerical self-consistent calculation confirms the existence of a swirling instability [34].

Through discrete and continuum models, we elucidated a novel swirling instability of arrays of elastic filaments, lending support to the hypothesis [14] that cytoplasmic streaming flows in *Drosophila* oocytes are tied to self-organization of the microtubule cytoskeleton. Further evidence for this hypothesis may come from genetic or other perturbations that explore the parameter space in Fig. 4(a). Future studies could shed light on the detailed mechanism involved in the untangling of the *Drosophila* oocyte cytoskeleton when it transitions to the vortical state, and the possibility of reproducing this transition *in vitro*. Last, this study highlights the role of active force dipoles in the self-organization of fluid-biopolymer systems [42–44].

We are indebted to Maik Drechsler and Isabel Palacios for sharing the data in Fig. 1 and to Vladimir Gelfand, Daniel St Johnston, and Stanislav Shvartsman for discussions on *Drosophila* streaming. This work was supported in part by ERC Consolidator Grant No. 682754 (E. L.), Wellcome Trust Investigator Grant No. 207510/Z/17/Z, Established Career Fellowship EP/M017982/1 from the Engineering and Physical Sciences Research Council, and the Schlumberger Chair Fund (R. E. G.). M. J. S. acknowledges the support of NSF Grant No. DMS-1620331.

## Supplementary Material

Supplementary_Material

Video_1

## Figures and Tables

**Fig. 1:**
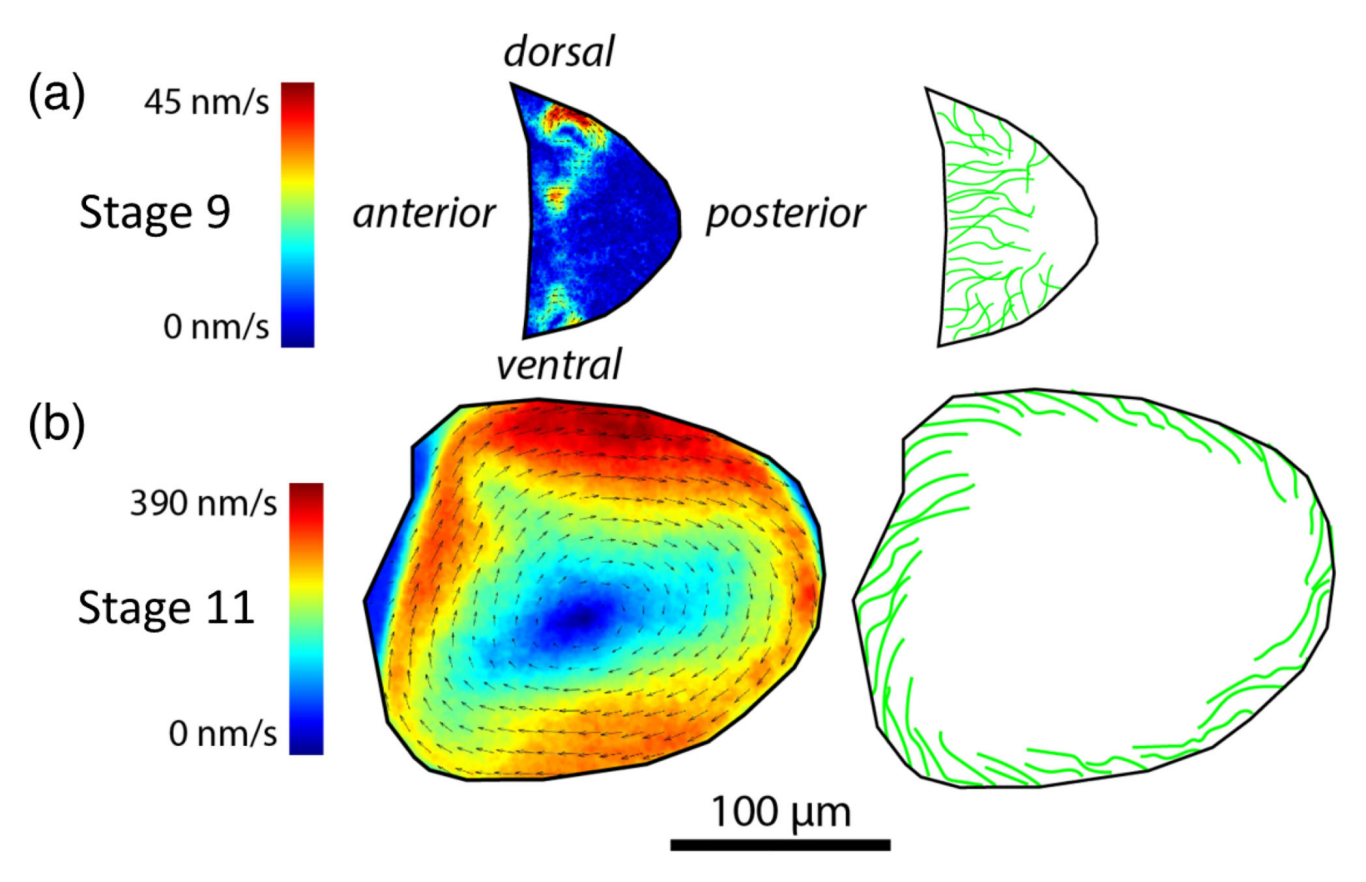
Cytoplasmic streaming in the *Drosophila* oocyte. The three-dimensional oocyte shape is approximately given by rotating the cross section about its anterior-posterior axis. (a) Experimental flow field [15] and schematic of the disordered swirling flows and microtubule organization in early stages of development. (b) Later flows organize into a single vortex as MTs lie parallel to the cell periphery.

**Fig. 2:**
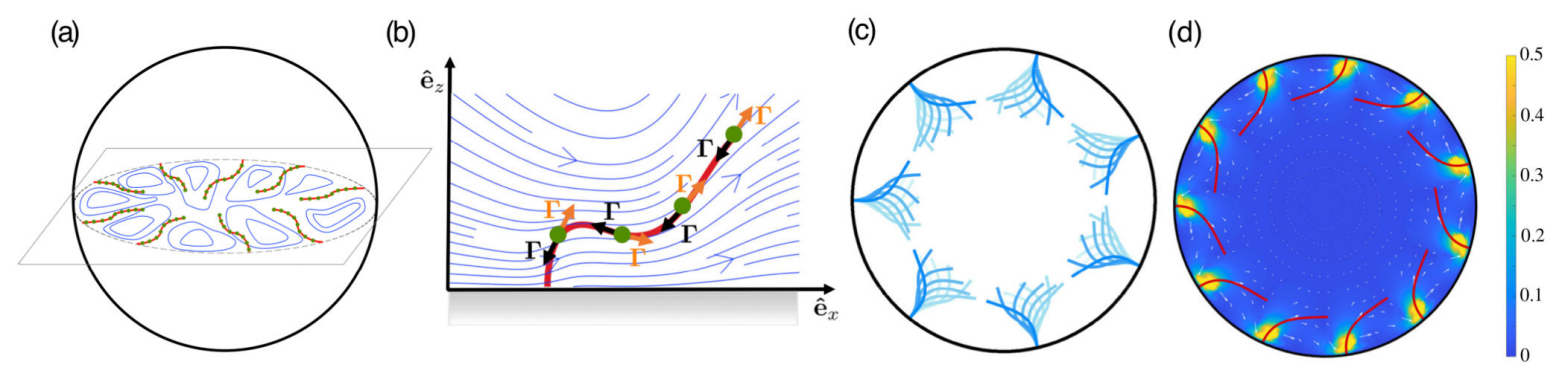
Discrete-filament computations. (a) *N* equally spaced filaments clamped at their attachment points, reach inward from a no-slip spherical shell. Each has a continuous distribution of tangential point forces (green) that (b) exert a force **Γ** on the fluid and an equal and opposite compressive force on the filament. Synchronous oscillations (*N* = 7, *σ* = 1700), (d) steady, bent configuration (*N* = 11, *σ* = 1100) and swirling flow field.

**Fig. 3:**
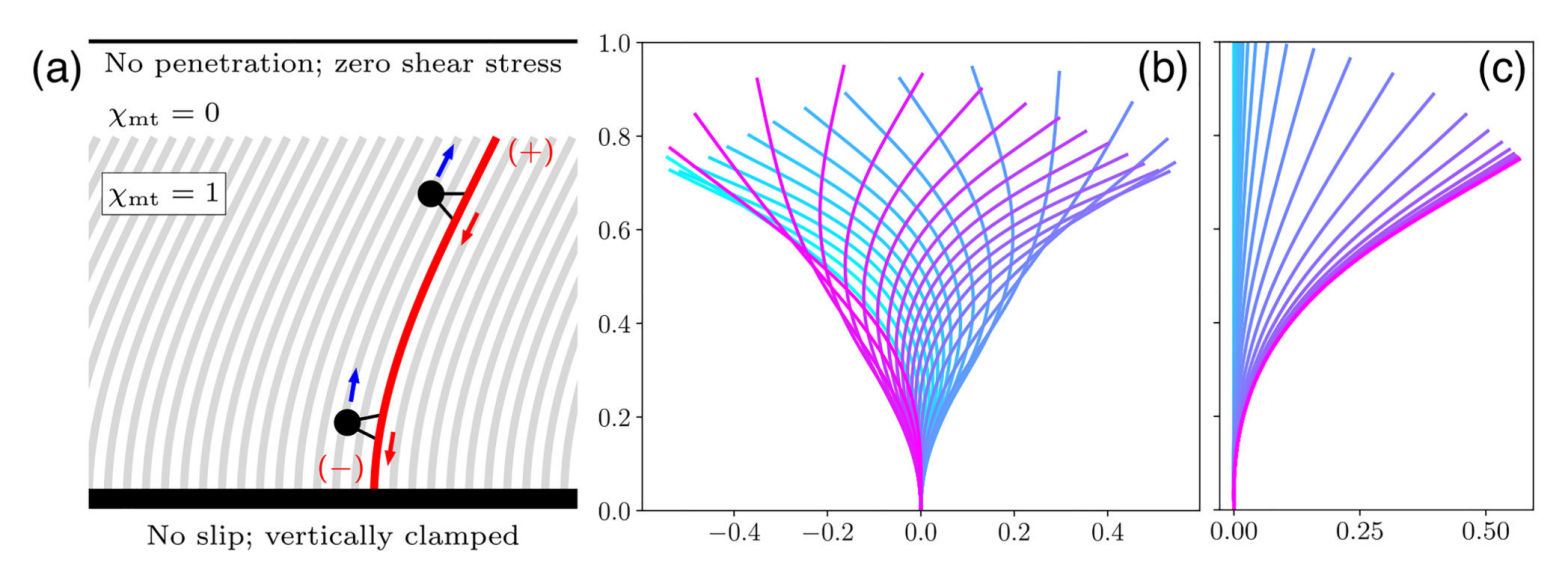
Continuum model in planar geometry. (a) Bi-infinite array of MTs, clamped vertically at a no-slip boundary. Results of computations at *ρ* = 4.65: (b) *σ* = 70 and (c) *σ* = 39. Colors denote time, from cyan (early) to pink (late).

**Fig. 4:**
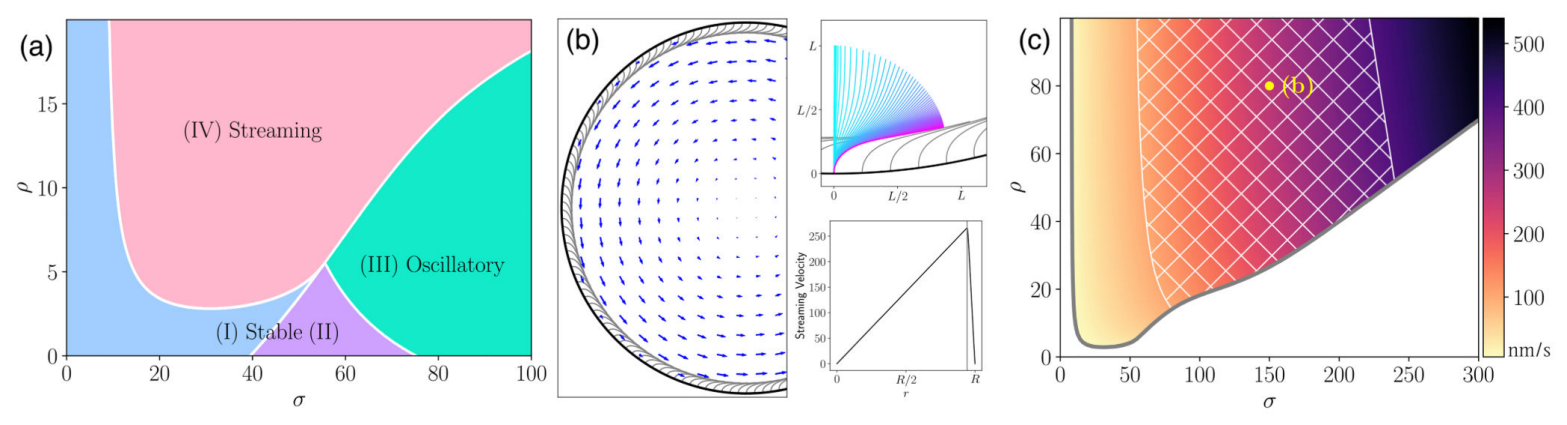
Continuum model in cylindrical geometry. (a) Results of linear stability analysis about the radially aligned state, with *R* = 5*L*. (b) Steady-state fiber deformations and velocity field for *σ* = 150 and *ρ* = 80. Density of visualized fibers corresponds to the physical density. The top inset shows deformed MTs and the dynamics of a representative one (see also the Supplemental Video [34]). The bottom inset shows the azimuthal velocity field as a function of *r*. (c) Dimensional streaming velocities in parameter space; hatched region is consistent with *in vivo* estimates of 100–400 nm/s. Yellow dot denotes simulation shown in (b).

**Fig. 5:**
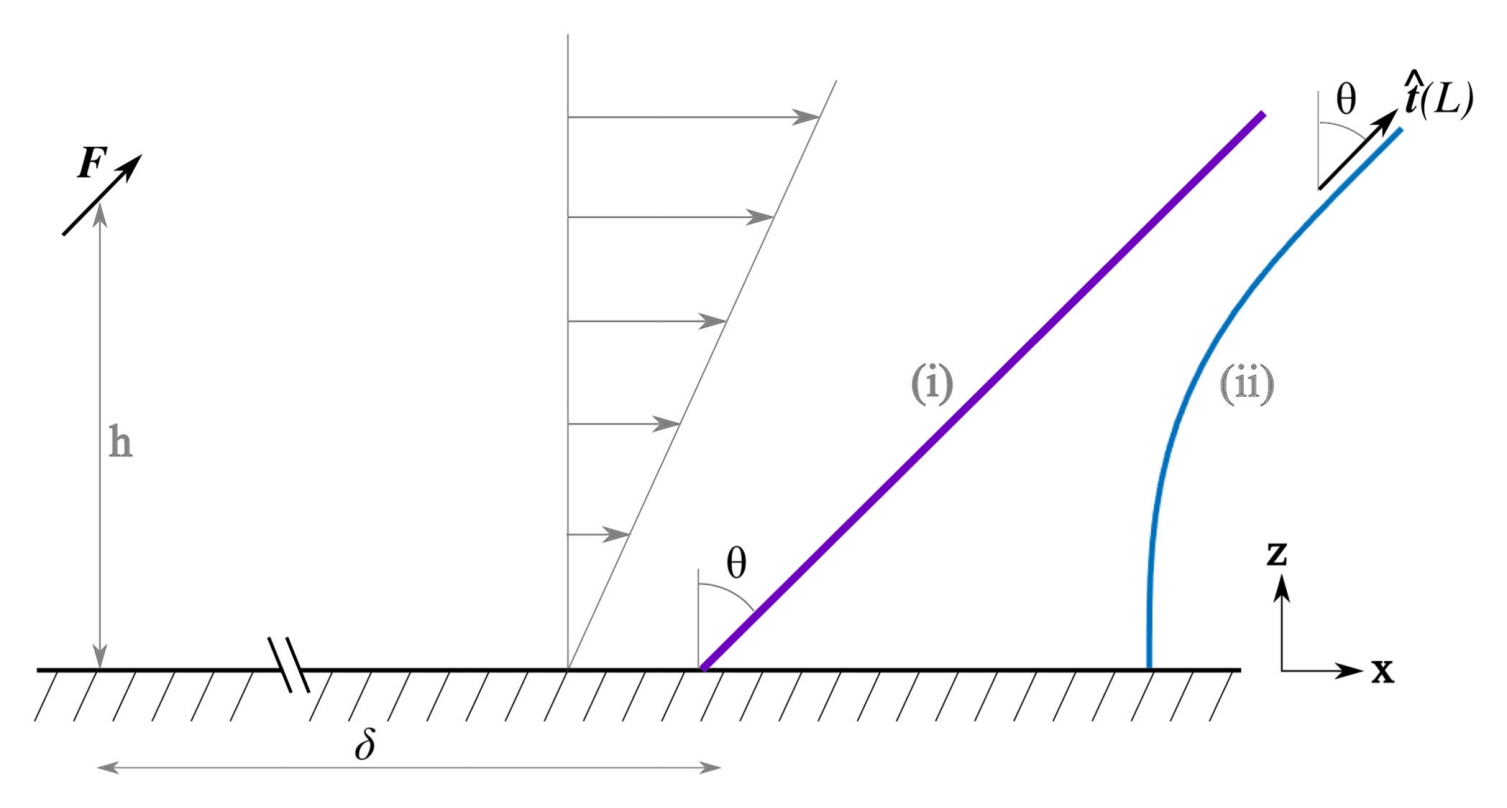
Self-consistent model. An upstream point force **F** parallel to the distal filament end produces shear flow that deflects the filament. Two variants: (i) rigid rod with a torsional spring at its base and (ii) a clamped elastic filament.

## References

[1] Needleman D, Shelley MJ (2019). The stormy fluid dynamics of the living cell. Phys Today.

[2] Bastock R, St Johnston D (2008). *Drosophila* oogenesis. Curr Biol.

[3] He L, Wang X, Montell DJ (2011). Shining light on Drosophila oogenesis: Live imaging of egg development. Curr Opin Genet Dev.

[4] Corti B (1774). Osservazioni Microscopische sulla Tremella e sulla Circolazione del Fluido in Una Pianta Acquajuola.

[5] Goldstein RE, van de Meent J-W (2015). A physical perspective on cytoplasmic streaming. Interface Focus.

[6] Gutzeit H, Koppa R (1982). Time-lapse film analysis of cytoplasmic streaming during late oogenesis of *Drosophila*. J Embryol Exp Morphol.

[7] Theurkauf W, Smiley S, Wong M, Alberts B (1992). Reorganization of the cytoskeleton during *Drosophila* oogenesis: Implications for axis specification and intercellular transport. Development.

[8] Theurkauf WE (1994). Premature microtubule-dependent cytoplasmic streaming in cappuccino and spire mutant oocytes. Science.

[9] Palacios IM, St Johnston D (2002). *Kinesin light chain-independent function of the kinesin heavy chain* in cyto-plasmic streaming and posterior localisation in the *Drosophila* oocyte. Development.

[10] Serbus LR, Cha BJ, Theurkauf WE, Saxton WM (2005). Dynein and the actin cytoskeleton control kinesin-driven cytoplasmic streaming in *Drosophila* oocytes. Development.

[11] Ganguly S, Williams LS, Palacios IM, Goldstein RE (2012). Cytoplasmic streaming in *Drosophila* oocytes varies with kinesin activity and correlates with the microtubule cytoskeleton architecture. Proc Natl Acad Sci USA.

[12] Drechsler M, Giavazzi F, Cerbino R, Palacios IM (2017). Active diffusion and advection in *Drosophila* oocytes results from the interplay of actin and microtubules. Nat Commun.

[13] Drechsler M, Lang LF, Al-Khatib L, Dirks H, Burger M, Schönlieb C-B, Palacios IM (2020). Optical flow analysis reveals that kinesin-mediated advection impacts the orientation of microtubules in the *Drosophila* oocyte. Mol Biol Cell.

[14] Monteith CE, Brunner ME, Djagaeva I, Bielecki AM, Deutsch JM, Saxton WM (2016). A mechanism for cytoplasmic streaming: Kinesin-driven alignment of microtubules and fast fluid flows. Biophys J.

[15] Palacios IM, Drechsler M (private communication), based on methods detailed earlier [11,16].

[16] Khuc Trong P, Guck J, Goldstein RE (2012). Coupling of Active Motion and Advection Shapes Intracellular Cargo Transport. Phys Rev Lett.

[17] Williams LS, Ganguly S, Loiseau P, Ng BF, Palacios IM (2014). The auto-inhibitory domain and the ATP-independent microtubule-binding region of kinesin heavy chain are major functional domains for transport in the *Drosophila* germline. Development.

[18] Khuc Trong P, Doerflinger H, Dunkel J, St Johnston D, Goldstein RE (2015). Cortical microtubule nucleation can organise the cytoskeleton of *Drosophila* oocytes to define the anteroposterior axis. eLife.

[19] Lu W, Lakonishok M, Serpenskaya AS, Kirchenbüechler D, Ling S-C, Gelfand VI (2018). Ooplasmic flow cooperates with transport and anchorage in *Drosophila* oocyte posterior determination. J Cell Biol.

[20] Gittes F, Mickey B, Nettleton J, Howard J (1993). Flexural rigidity of microtubules and actin filaments measured from thermal fluctuations in shape. J Cell Biol.

[21] Visscher K, Schnitzer MJ, Block SM (1999). Single kinesin molecules studied with a molecular force clamp. Nature (London).

[22] Herrmann G, Bungay RW (1964). On the stability of elastic systems subjected to nonconservative forces. J Appl Mech.

[23] Bayly PV, Dutcher SK (2016). Steady dynein forces induce flutter instability and propagating waves in mathematical models of flagella. J R Soc Interface.

[24] De Canio G, Lauga E, Goldstein RE (2017). Spontaneous oscillations of elastic filaments induced by molecular motors. J R Soc Interface.

[25] Ling F, Guo H, Kanso E (2018). Instability-driven oscillations of elastic microfilaments. J R Soc Interface.

[26] Young Y-N, Shelley MJ (2007). Stretch-Coil Transition and Transport of Fibers in Cellular Flows. Phys Rev Lett.

[27] Kantsler V, Goldstein RE (2012). Flucutations, Dynamics, and the Stretch-Coil Transition of Single Actin Filaments in Extensional Flows. Phys Rev Lett.

[28] Stein DB, Shelley MJ (2019). Coarse graining the dynamics of immersed and driven fiber assemblies. Phys Rev Fluids.

[29] Lu W, Winding M, Lakonishok M, Wildonger J, Gelfand VI (2016). Microtuble-microtubule sliding by kinesin-1 is essential for normal cytoplasmic streaming in *Drosophila* oocytes. Proc Natl Acad Sci USA.

[30] Keller JB, Rubinow SI (1976). Slender-body theory for slow viscous flow. J Fluid Mech.

[31] Gray J, Hancock GJ (1955). The propulsion of sea-urchin spermatozoa. J Exp Biol.

[32] Tornberg AK, Shelley MJ (2004). Simulating the dynamics and interactions of flexible fibers in Stokes flows. J Comput Phys.

[33] Goldstein RE, Langer SA (1995). Nonlinear Dynamics of Stiff Polymers. Phys Rev Lett.

[34] http://link.aps.org/supplemental/10.1103/PhysRevLett.126.028103.

[35] De Canio G (2018). Motion of filaments induced by molecular motors: From individual to collective dynamics.

[36] Maul C, Kim S (1994). Image systems for a Stokeslet inside a rigid spherical container. Phys Fluids.

[37] Goldstein RE (2015). Green algae as model organisms for biological fluid dynamics. Annu Rev Fluid Mech.

[38] Niedermayer T, Eckhardt B, Lenz P (2008). Synchronization, phase locking, and metachronal wave formation in ciliary chains. Chaos.

[39] Man Y, Kanso E (2020). Multisynchrony in Active Microfilaments. Phys Rev Lett.

[40] Yotsuyanagi Y (1953). Recherches sur les phénomenès moteurs dans les fragments de protoplasme isolés. I. Mouvement rotatoire et le processus de son apparition. Cytologia.

[41] Wioland H, Woodhouse FG, Dunkel J, Kessler JO, Goldstein RE (2013). Confinement Stabilizes a Bacterial Suspensions into a Spiral Vortex. Phys Rev Lett.

[42] Saintillan D, Shelley MJ (2008). Instabilities and Pattern Formation in Active Particle Suspensions: Kinetic Theory and Continuum Simulations. Phys Rev Lett.

[43] Saintillan D, Shelley MJ, Zidovska A (2018). Extensile motor activity drives coherent motions in a model of interphase chromatin. Proc Natl Acad Sci USA.

[44] Woodhouse FG, Goldstein RE (2012). Spontaneous Circulation of Confined Active Suspensions. Phys Rev Lett.

[45] Man Y, Kanso E (2019). Morphological transitions of axially-driven microfilaments. Soft Matter.

[46] Nazockdast E, Rahimian A, Zorin D, Shelley MJ (2017). A fast platform for simulating semi-flexible fiber suspensions applied to cell mechanics. J Comput Phys.

[R47] [47]Differentiation of (2) yields an equivalent equation for the tangent-vector field **r**_*s*_ [28], which we have found to be numerically more stable.

[48] Landau LD, Lifshitz EM (1970). Theory of Elasticity.

[49] Goldstein RE, Warren PB, Ball RC (2012). Shape of a Ponytail and the Statistical Physics of Hair Fiber Bundles. Phys Rev Lett.

[50] Loiseau P, Davies R, Williams LS, Mishima M, Palacios M (2010). *Drosophila* PAT1 is required for kinesin-1 to transport cargo and to maximize its motility. Development.

[51] Brumley DR, Wan KY, Polin M, Goldstein RE (2014). Flagellar synchronization through direct hydrodynamic interactions. eLife.

[52] Blake JR (1971). A note on the image system for a stokeslet in a no-slip boundary. Math Proc Cambridge Philos Soc.

[53] Thomazo J-B, Lauga E, Le Réevérend B, Wandersman E, Prevost AM (2020). Collective stiffening of soft hair assembles. Phys Rev E.

[54] Kimura K, Mamane A, Sasaki T, Sato K, Takagi J, Niwayama R, Hufnagel L, Shimamoto Y, Joanny JF, Uchida S, Kimura A (2017). Endoplasmic-reticulum-mediated microtubule alignment governs cytoplasmic streaming. Nat Cell Biol.

